# Elevated expression of a minor isoform of *ANK3* is a risk factor for bipolar disorder

**DOI:** 10.1038/s41398-018-0175-x

**Published:** 2018-10-08

**Authors:** Timothy Hughes, Ida E. Sønderby, Tatiana Polushina, Lars Hansson, Asbjørn Holmgren, Lavinia Athanasiu, Christian Melbø-Jørgensen, Sahar Hassani, Louise K. Hoeffding, Stefan Herms, Sarah E. Bergen, Robert Karlsson, Jie Song, Marcella Rietschel, Markus M. Nöthen, Andreas J. Forstner, Per Hoffmann, Christina M. Hultman, Mikael Landén, Sven Cichon, Thomas Werge, Ole A. Andreassen, Stephanie Le Hellard, Srdjan Djurovic

**Affiliations:** 1 Department of Medical Genetics, Oslo University Hospital, Oslo, Norway; 2 NORMENT, KG Jebsen Centre for Psychosis Research, Institute of Clinical Medicine, University of Oslo, Oslo, Norway; 3 Department of Clinical Science, NORMENT, KG Jebsen Centre for Psychosis Research, University of Bergen, Bergen, Norway; 4 Dr Einar Martens Research Group for Biological Psychiatry, Centre for Medical Genetics and Molecular Medicine, Haukeland University Hospital, Bergen, Norway; 5 Institute of Biological Psychiatry, Mental Health Centre Sct. Hans, Copenhagen University Hospital, Roskilde, Denmark; 6 iPSYCH, The Lundbeck Foundation Initiative for Integrative Psychiatric Research, Copenhagen, Denmark; 7 Department of Biomedicine, Human Genomics Research Group, University of Basel, Basel, Switzerland; 8 Institute of Human Genetics, University of Bonn, Bonn, Germany; 9 Department of Genomics, Life & Brain Center, University of Bonn, Bonn, Germany; 10 Department of Medical Epidemiology and Biostatistics, Karolinska Institutet, Stockholm, Sweden; 11 Department of Genetic Epidemiology in Psychiatry, Central Institute of Mental Health, Medical Faculty Mannheim/Heidelberg University, Mannheim, Germany; 12 Department of Psychiatry (UPK), University of Basel, Basel, Switzerland; 13 Institute of Medical Genetics and Pathology, University Hospital Basel, Basel, Switzerland; 14 Institute of Neuroscience and Physiology, The Sahlgrenska Academy at Gothenburg University, Gothenburg, Sweden; 15 Institute of Neuroscience and Medicine (INM-1), Research Center Juelich, Juelich, Germany; 16 Department of Clinical Medicine, University of Copenhagen, Copenhagen, Denmark; 17 NORMENT, KG Jebsen Centre for Psychosis Research, Division of Mental Health and Addiction, Oslo University Hospital, Oslo, Norway

## Abstract

*Ankyrin-3 (ANK3)* is one of the few genes that have been consistently identified as associated with bipolar disorder by multiple genome-wide association studies. However, the exact molecular basis of the association remains unknown. A rare loss-of-function splice-site SNP (rs41283526*G) in a minor isoform of *ANK3* (incorporating exon ENSE00001786716) was recently identified as protective of bipolar disorder and schizophrenia. This suggests that an elevated expression of this isoform may be involved in the etiology of the disorders. In this study, we used novel approaches and data sets to test this hypothesis. First, we strengthen the statistical evidence supporting the allelic association by replicating the protective effect of the minor allele of rs41283526 in three additional large independent samples (meta-analysis *p*-values: 6.8E–05 for bipolar disorder and 8.2E–04 for schizophrenia). Second, we confirm the hypothesis that both bipolar and schizophrenia patients have a significantly higher expression of this isoform than controls (*p*-values: 3.3E–05 for schizophrenia and 9.8E–04 for bipolar type I). Third, we determine the transcription start site for this minor isoform by Pacific Biosciences sequencing of full-length cDNA and show that it is primarily expressed in the corpus callosum. Finally, we combine genotype and expression data from a large Norwegian sample of psychiatric patients and controls, and show that the risk alleles in *ANK3* identified by bipolar disorder GWAS are located near the transcription start site of this isoform and are significantly associated with its elevated expression. Together, these results point to the likely molecular mechanism underlying *ANK3*´s association with bipolar disorder.

## Introduction

Bipolar disorder (BD) is a severe psychiatric pathology with a lifetime prevalence rate of 1–2%^1^. Although clearly polygenic in nature, BD’s high heritability has enabled well-powered genome-wide association studies (GWAS) to reveal part of the genetic architecture of the disorder by identifying common genetic variants that contribute to BD risk^2^. However, most associated variants are located in non-coding regions and are typically, but not always^3^, difficult to interpret functionally. Thus, although associated variants may provide relatively strong evidence of a gene’s implication in the etiology, they typically shed little light on the underlying molecular mechanisms^4,5^.

The case of the *ANK3* gene (encoding ankyrin-G) illustrates this well. It was the first gene to be implicated in a BD GWAS study^6^ and the association has been confirmed by multiple subsequent studies^7–10^, yet it remains unclear which specific gene isoform mediates the association, whether it is a lowered or elevated level of functional protein which is a risk factor, or in which cell type the relevant isoform is expressed.

**Fig. 1:**
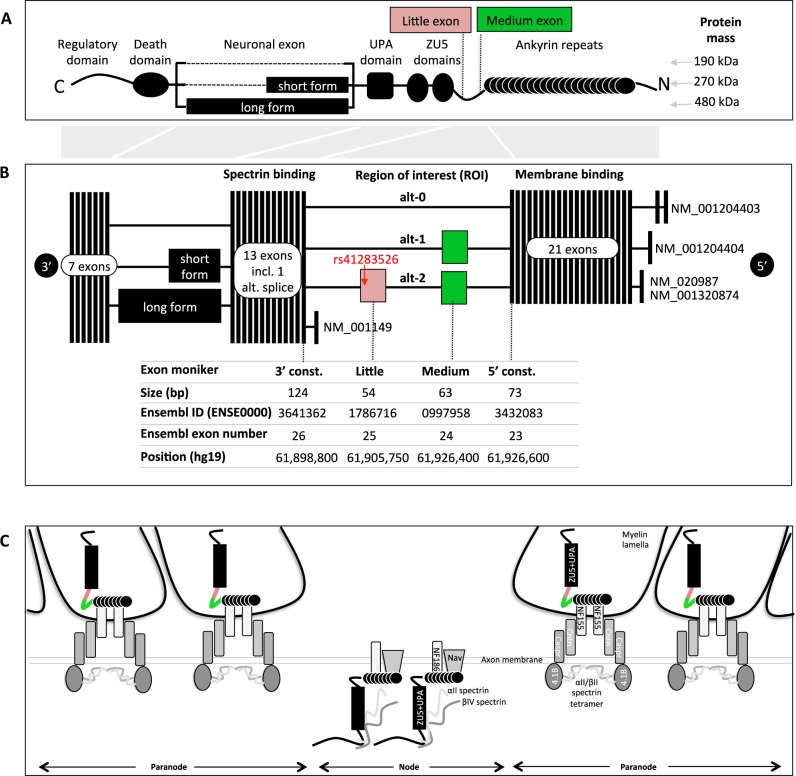
*ANK3* gene and protein structure. **a** Domain structure of the main isoforms of the Ankyrin-G protein with an orientation that matches the exonic structures in **b** and with the position of the “little” exon (ENSE00001786716) highlighted. The term “neuronal” exon is historical and perhaps a misnomer since recent data suggests that the short form of the “neuronal” exon is expressed in oligodendrocytes. **b** Summary of the exonic structure of *ANK3* based on the five transcripts in the RefSeq database. Gray shading between **a** and **b** depicts the exon to domain mapping. **c** *ANK3’s* interaction partners at the node and paranode in the central nervous system^14,45,46^. Cntn contactin, Caspr contactin-associated protein, NF neurofascin, Nav voltage-gated sodium channel, 4.1B protein 4.1B. Little exon in pink, medium exon in green

Ankyrin-G is a large scaffolding adapter protein consisting of two major domains: the ankyrin repeats which bind membrane proteins, and the ZU5 and UPA domains which bind β-spectrin^11,12^. It has several alternatively spliced exons, the largest of which has historically been referred to as the “neuronal” exon and can be spliced in three different ways^11^, resulting in big differences in total protein mass (Fig. 1a). Different versions of these large isoforms have been detected in neurons at the nodes of Ranvier^13^, the axon initial segment and dendritic spines^13^, and in oligodendrocytes at the paranodal axoglial junction^14,15^ (Fig. 1c). There are also other smaller alternatively spliced exons (Fig. 1b) and the RefSeq database lists five transcripts^16^, but the Ensembl database^17^ and our own data suggest that this is not an exhaustive list.

We recently identified, in one of these alternatively spliced exons (Ensembl exon ID: ENSE00001786716), a rare loss-of-function splice-site variant (rs41283526*G), which is strongly protective of both BD and schizophrenia (SCZ)^18^. We name this exon the “little” exon in reference to its short length (Fig. 1b). The variant has a large and statistically significant effect size, but probably only plays a minor role in the genetic architecture of the disease, since its allelic frequency is very low (1–2% in European populations and unobserved in Asian or African populations)^18^. However, it does provide valuable insight into the molecular mechanism since a protective loss-of-function suggests that an elevated expression of this specific gene isoform may be a risk factor.

The present study has four tightly related aims. First, to further test the protective effect of the loss-of-function allele of rs41283526 in new independent samples to better estimate the odds ratio. Second, to directly test whether the expression of the *ANK3* transcript incorporating exon ENSE00001786716 is elevated in cases. Third, to determine the transcription start site (TSS) and cell-type specificity of this isoform. And, fourth, to investigate the relationship between the current genome-wide associated SNPs and the expression of this isoform.

## Materials and methods

### Association replication and meta-analysis

In the present study, we performed allelic association tests for rs41283526 in seven independent samples and a meta-analysis. Three of these samples were used in our previous work^18^, while four of them are new.

The samples from previous work are Norwegian (BD and SCZ) and Swedish (BD). The Swedish BD data is used unchanged (2286 cases, 5412 controls), but the Norwegian samples are a merging of exome chip and TaqMan genotyped data sets. As a result, we have one Norwegian BD data set (504 cases), one Norwegian SCZ data set (411 cases), and one Norwegian control data set (369 controls).

We set two requirements for the new samples: (1) rs41283526 needs to be present at as high a frequency as possible, thus we focus on North-European samples where the allele frequency ranges between 1.2 and 3.1 percent (Table S2); (2) samples must be directly genotyped for this functional variant as the quality of imputation of SNPs in this frequency range is poor^19^. We identified four cohorts that fulfill these criteria: Danish BD (193 cases and 696 controls), German BD (895 cases and 2367 controls), Swedish SCZ (4822 cases and 6088 controls), and Danish SCZ (1872 cases and 1091 controls). All studies obtained informed consent from participants and obtained approval of protocols and procedures from their local Ethics Committees (Sample details in Table S3).

We used PLINK^20^ to test for Hardy–Weinberg equilibrium (Table S1) and for allelic association on all samples (–fisher option). Fisher one-tailed tests were performed in R (Table 1A). We did not perform genotypic tests because of the low frequency of the SNP.

**Table 1A:** Allelic association tests for rs41283526*G performed in PLINK and R

Samples		Numbers		Allele frequency				*p*-value	
		Cases	Controls	Cases	Controls	OR	Std. err.	Fisher (2-sided)	Fisher (1-sided)
BD	Norway	504	369	0.008	0.031	0.25	0.41	3.6E–04	2.8E–04
	Sweden	2286	5412	0.012	0.016	0.72	0.16	4.0E–02	2.1E–02
	Denmark^a^	193	696	0.008	0.016	0.49	0.62	3.3E–01	1.7E–01
	Germany^a^	895	2367	0.006	0.012	0.51	0.33	4.0E–02	2.1E–02
SCZ	Norway	411	369	0.005	0.031	0.15	0.54	5.4E–05	4.6E–05
	Sweden^a^	4822	6088	0.014	0.018	0.81	0.11	6.6E–02	3.3E–02
	Denmark^a^	1872	1091	0.009	0.014	0.64	0.25	8.7E–02	5.0E–02

^a^Indicates new replication data sets

In addition to being analyzed separately, the four new samples presented here were combined with the three samples from previous work to give a total of seven allelic association tests (four BD and three SCZ) and used in a meta-analysis. We use METAL^21^ to calculate a signed *Z*-score test: *Z*-scores for each allele are combined across samples in a weighted sum, with weights proportional to the square-root of the sample size for each study. We used PLINK^20^ to calculate both fixed-effect and random-effect models (--meta-analysis option). Heterogeneity statistics were computed in PLINK (*Q* and *I*^2^) and METAL (I^2^ heterogeneity test).

### BrainSpan developmental transcriptome

We analyzed *ANK3* exon expression in the developmental transcriptome data set from BrainSpan^22^. This is a RNA-seq data set from the brains of 38 individuals with ages ranging from 5 weeks post-conception to 40 years old where tissue samples were taken from 8 to 16 structures of each brain. This data set provides a quantitation of expression levels of each exon of *ANK3* in each structure at different points in human brain development. We plotted this data in Fig. 3a and performed a Wilcoxon one-sided test of higher expression of the “little” exon in individuals aged 11 or older (vs. individuals aged 0 to 10 years old) in 16 different brain regions (Table S4).

### Differential expression in blood

A total of 919 blood samples for gene expression analysis were collected (Table S5) using Tempus Blood RNA Tubes (Life Technologies Corporation, Carlsbad, CA, USA). RNA extraction and cDNA generation was performed as described in detail in the supplementary materials. Expression of the alt-2 splice pattern (Fig. 1b) was measured by RT-qPCR on the ABI PRISM 7900HT Real-Time PCR System (Life Technologies Corporation, Carlsbad, CA, USA), using a SYBR green assay (see Supplementary materials). We performed one-sided *t* test of higher expression in cases than controls for each of the diagnostic groups (both including and excluding carriers of rs41283526*G) (Table 2; Fig. 3b). Diagnostic groups are healthy controls (CTRL), schizophrenia broad definition (SCZ), BD type I (BDI), BD type II (BDII), and major depression and psychosis not otherwise specified (MIX).

**Table 2:** Test of elevated expression of alt-2 splice pattern in blood by diagnostic group

Genotype	Group	Samples	Expression	FC	*t* test
		(count)	(mean)		(*p*-value)
All genotyped samples					
	CTRL	269	0.982	NA	NA
	MIX	123	1.007	1.026	1.5E–01
	SCZ	309	1.062	1.082	3.3E–05
	BDNOS	19	1.061	1.081	1.3E–01
	BD I	140	1.064	1.084	9.8E–04
	BDII	59	0.946	0.964	8.4E–01
	BDI & SCZ	449	1.063	1.083	7.6E–06
Excl. carriers of rs41283526*G					
	CTRL	256	1.004	NA	NA
	MIX	120	1.014	1.010	3.3E–01
	SCZ	309	1.062	1.058	1.3E–03
	BDNOS	19	1.061	1.058	2.1E–01
	BD I	138	1.073	1.069	3.6E–03
	BDII	59	0.946	0.943	9.5E–01
	BDI & SCZ	447	1.065	1.062	2.8E–04

One-sided tests of elevated expression in cases vs. controls (for each diagnostic group)

FC: fold change (diag/ctrl)

### *ANK3* isoforms in human brain

#### Brain regions expressing alt-2

There are three splice patterns in the region of interest (genetic ROI), as illustrated in Fig. 1b: (1) alt-0 which incorporates neither the “little” nor the “medium”; (2) alt-1 that incorporates the “medium” exon; (3) alt-2 which incorporates both the “medium” and the “little” exon. In order to determine whether there are differences between brain regions in the expression of the different splice patterns, we obtained total RNA pools from seven different brain regions (Clontech Laboratories Inc, Mountain View, California) and generated cDNA (Table S6). We designed primers in the “5′ constitutive” exon and the “3′ constitutive” exon (Fig. 1b) and we performed a PCR amplification of the region containing the “little” exon. The amplified products were subjected to capillary electrophoresis on the 3730xl DNA Analyzer (Applied Biosystems, Foster City, California) and splice patterns were detected on the GeneMapper 5 software (Applied Biosystems). The data clearly show that the alt-1 and alt-2 splice patterns are more highly expressed in cerebral cortex and corpus callosum (Table S7).

#### Active transcription start sites

We next sought to determine which are the active transcription start sites in these two tissues where alt-1 and alt-2 are expressed. We generated full-length cDNA from 1 µg of total RNA, using the SMARTer® PCR cDNA Synthesis Kit (Takara Bio USA, Inc., Mountain View, CA, USA). Target-specific products were generated directly from first-strand cDNA products, using the QIAGEN LongRange PCR Kit (QIAGEN, Hilden, Germany) and four different forward primers placed in each of the four first exons of *ANK3* (Fig. 1b) and one common reverse primer in the 3’ UTR. Finally, peak detection was performed with the Agilent DNA 12000 Kit on the 2100 Bioanalyzer system (Agilent Technologies, Santa Clara, CA, USA).

We obtained no amplification from the TSSes of NM_001204403 or NM_001149 and multiple peak sizes in the expected ranges for NM_001204404 and NM_020987 (Table S8). The large differences in product size (varying from 6 to 12 kbp) are due to the alternative splicing of the large “neuronal” exon (Fig. 1a).

#### Pacific Biosciences sequencing

We aimed to determine from which TSS the different splice patterns of the genetic ROI are expressed and their relative abundance (in corpus callosum and cerebral cortex). Since we intended to determine the exonic structures while preserving relative abundances, we avoided length amplification bias by generating shorter amplification products: we use the full-length cDNA previously generated and performed target-specific PCR using the forward primers in the first exon of NM_001204404 and NM_020987 (as these are the active TSSs) and a reverse primer three exons downstream of the ROI (rather than in the 3’ UTR). For each brain region, the two amplifications were pooled on equal volumes and sample preparation for Pacific Biosciences sequencing was performed with the SMRTbell Template Prep Kit 1.0 followed by sequencing on one flow cell of the Pacific BioSciences RS II (Menlo Park, CA, USA). We obtained ~700k subreads for each brain region library (Table S9 and S10).

We removed any reads that were not in the expected size range of 2.7 to 3.3 kbp and where mean read quality was below 85 percent. We determined the exonic content of each read by searching for the sequence of the exons of interest using the BBDUK software (http://jgi.doe.gov/data-and-tools/bbtools/) with kmer length of 18 bp and edit distance of 2 (necessary because of the high error rate in Pacific Biosciences data). In both brain regions, there were a few dominant splice patterns present at 1% or more and a large number present at <1%. Of the splice patterns present at <1%, some were due to a failure to correctly detect an exon sequence due to the high error rate of the SMRT system, whereas others may reflect molecules that are present at very low concentrations. For any splice pattern present in one of the brain regions at 1% or more, we summarized the percentage of subreads corresponding to this pattern in each of the brain regions (Fig. 4a).

### Effect of SNPs of interest on expression

We wished to determine whether there was any correlation between 28 SNPs of interest and the expression measures of the “little” exon in blood. We defined SNPs of interest as the 22 SNPs that were genome-wide significant in the PGC BD GWAS study^7^ as well as the SNP with the largest OR in the same study (rs16914810), rs41283526, and four other SNPs that are in proximity to it and in strong LD with it. We obtained summary statistics for the PGC BD GWAS from the Ricopili tool (https://data.broadinstitute.org/mpg/ricopili/). Norwegian samples were directly genotyped and imputed as previously described^23^. Haplotypes were inferred from the 1167 genotyped Norwegian samples using Haploview^24,25^, and haploblocks were determined using the 4 gametes rule^26^ (with the requirement that the 4th gamete be observed at a frequency above 1%). *D*’ and *r*^2^ were computed using PLINK^20^ with –ld option for 1167 samples and plotted in R (Fig. 4c).

In the 705 Norwegian samples for which we had both genotypes and measures of expression of alt-2 in blood, we computed one-sided Wilcoxon tests of higher expression of the little exon in minor allele carriers vs. non-carriers for each of the SNPs of interest (Table 3). We used the Wilcoxon test because of the low frequency of the minor alleles at these sites. We also performed the same test with the carriers of rs41283526*G excluded from the analysis (reducing the number of samples to 691) in order to exclude the known strong effect of this variant on the expression of the “little” exon^18^.

**Table 3:** Summary GWAS statistics from the PGC bipolar disorder GWAS at 28 loci and differential expression of the “little” exon by genotype

#	Single nucleotide polymorphism					PGC 2011 BD GWAS		Norwegian sample (eQTL test)				
	dbSNP RSID	Position (hg19: chr10)	Major allele	Minor allele	G1K European MAF	*P*-value	OR (minor)	MAF	All samples (n=705)		Excl.carriers of rs41283526*G (n=691)	
									FC	*p*-value	FC	*p*-value
1	rs2288359^a^	61902023	C	**T**	0.059	1.2E–01	1.09	0.064	1.09	**5.3E–04**	1.08	**1.2E–03**
2	rs41283526^a^	61905727	A	G	0.016	3.6E–04^b^	0.25^b^	0.013	0.54	1.2E–08	NA^c^	NA^c^
3	rs12357972^a^	61907907	T	**C**	0.048	5.5E–03	1.16	0.051	1.08	**3.0E–03**	1.07	**5.8E–03**
4	rs16914810	61913414	T	**C**	0.047	2.1E–02	1.98	0.05	1.08	**2.0E–03**	1.07	**4.0E–03**
5	rs2393614^a^	61915955	C	T	0.319	9.6E–02	1.05	0.358	1.04	**2.5E–02**	1.02	8.0E–02
6	rs10509125^a^	61926866	A	**C**	0.394	2.5E–02	1.06	0.429	1.08	**4.9E–05**	1.06	**6.3E–04**
7	rs10761473	62060382	C	G	0.078	5.6E–09	1.36	0.057	1.01	3.9E–01	1.00	5.1E–01
8	rs10509129	62071041	G	T	0.047	5.8E–09	1.36	0.041	1.04	1.3E–01	1.03	1.9E–01
9	rs1380459	62097331	C	T	0.070	3.3E–09	1.36	0.052	1.03	1.2E–01	1.02	1.8E–01
10	rs10994308	62098952	G	A	0.070	3.8E–09	1.36	0.052	1.03	1.2E–01	1.02	1.8E–01
11	rs10821736^a^	62105053	C	T	0.070	1.3E–09	1.36	0.052	1.04	8.6E–02	1.03	1.3E–01
12	rs10821745^a^	62136206	T	G	0.069	2.3E–09	1.35	0.054	1.04	5.7E–02	1.03	9.2E–02
13	rs10994322^a^	62136279	C	T	0.069	6.7E–09	1.35	0.054	1.04	5.7E–02	1.03	9.2E–02
14	rs4948412	62146576	T	C	0.068	5.3E–09	1.35	0.054	1.04	5.7E–02	1.03	9.2E–02
15	rs3808943	62151015	C	T	0.069	4.7E–09	1.35	0.056	1.04	5.8E–02	1.03	9.4E–02
16	rs10821748	62152938	G	C	0.071	4.4E–08	1.32	0.054	1.04	5.7E–02	1.03	9.2E–02
17	rs12416380	62156154	A	G	0.069	7.5E–09	1.35	0.054	1.04	5.7E–02	1.03	9.2E–02
18	rs4948417^a^	62161618	A	G	0.068	5.5E–09	1.35	0.054	1.04	5.7E–02	1.03	9.2E–02
19	rs10994336^a^	62179812	C	T	0.073	4.0E–09	1.35	0.052	1.04	9.8E–02	1.03	1.5E–01
20	rs10994338^a^	62181128	C	T	0.073	6.7E–09	1.34	0.052	1.04	9.8E–02	1.03	1.5E–01
21	rs9633553	62274737	T	G	0.075	5.5E–10	1.35	0.054	1.05	7.0E–02	1.04	1.1E–01
22	rs10994397^a^	62279124	C	T	0.075	5.5E–10	1.35	0.054	1.05	7.0E–02	1.04	1.1E–01
23	rs12412135	62282834	C	**T**	0.094	4.2E–08	1.27	0.074	1.05	**1.5E–02**	1.05	**1.9E–02**
24	rs1938540	62294814	C	**T**	0.079	1.9E–09	1.33	0.054	1.06	**2.5E–02**	1.05	**4.3E–02**
25	rs10821792^a^	62298616	C	**T**	0.079	2.0E–09	1.32	0.054	1.06	**2.5E–02**	1.05	**4.3E–02**
26	rs1938526	62300383	A	**G**	0.079	1.9E–09	1.32	0.054	1.06	**2.5E–02**	1.05	**4.3E–02**
27	rs10994415	62322034	T	C	0.074	7.0E–10	1.31	0.075	1.04	8.1E–02	1.03	1.4E–01
28	rs2154393	62326687	C	T	0.074	3.7E–09	1.29	0.076	1.04	7.2E–02	1.03	1.3E–01

Fold change (FC) is the ratio of the mean “little” exon expression for minor allele carriers to the mean for non-carriers. Tests are one-sided Wilcoxon test of higher expression of the little exon in minor allele carriers vs. non-carriers (with the exception of rs41283526, where we test lower expression in carriers)

*p*-values are uncorrected for multiple testing and sites significant at 5% are highlighted in bold

Samples used in the differential expression tests (eQTL test) are either all samples for which genotypes and expression are available (*n* = 705) or excluding those carrying the minor allele at rs41283526 (*n* = 691)

MAF in Norwegian sample is based on 1167 genotyped samples. Horizontal lines delineate haploblocks as determined by four gametes method on the Norwegian sample

*MAF* minor allele frequency, *G1K* 1000 genomes project (503 European individuals in G1K)

^a^Indicates directly genotyped SNP (as opposed to imputed)

^b^SNP minor allele frequency was too low to be genotyped or imputed with confidence in PGC GWAS. Instead, we include here the results for the Norwegian BD sample for comparison with the PGC ORs

^c^Not available because of the exclusion of samples carrying rs41283526*G

## Results

### Association replication and meta-analysis

The sample-specific allelic association tests for rs41283526*G have protective ORs in all four samples: 0.49 (BD Denmark), 0.51 (BD Germany), 0.81 (SCZ Sweden), 0.64 (SCZ Denmark). In our previous work, we established that rs41283526*G has a protective effect, thus the one-tailed Fisher test is the relevant metric for replication and it shows that the association is replicated in three of the four new independent samples: the Germany BD (*p*-value = 2.1E–02), Sweden SCZ (*p*-value = 3.3E–02), and Denmark SCZ (*p*-value = 5.0E–02) samples (Table 1A).

All three meta-analysis two-sided tests (fixed effects, random effects, and weighted *Z*-score) were all nominally significant in both BD and SCZ groups (Table 1B). Heterogeneity metrics show that there is considerable heterogeneity between the samples, thus indicating that the random effects model probably provides the most reliable estimate of the odds ratio BD (0.5), SCZ (0.53), and 0.54 (BD & SCZ).

**Table 1B:** Meta-analysis for rs41283526*G performed in PLINK and METAL

Meta-analysis	Fixed effects		Random effects		Weighted *Z*-score		Heterogeneity		
	OR	*p*-value	OR	*p*-value	Direction	*p*-value	*Q*	*I* ^2^	*p*-value
**BD**	0.60	1.2E–04	0.50	5.1E–03	----	6.8E–05	0.10	52.20	9.9E–02
**SCZ**	0.74	2.5E–03	0.53	5.4E–02	---	8.2E–04	0.01	79.10	8.4E–03

In the weighted *Z*-score analysis, weights are set as recommend to the effective sample size: *N*_eff_ = 4/(1/*N*_cases_ + 1/*N*_ctrls_)

**Fig. 2:**
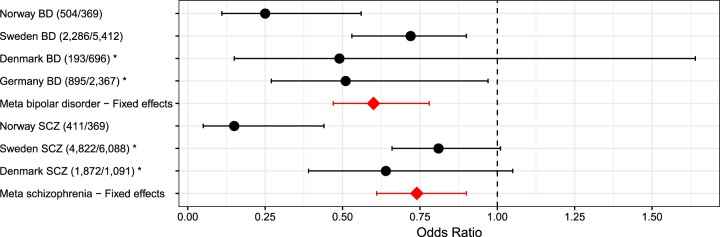
OR confidence intervals for rs41283526 in bipolar disorder and schizophrenia. Odds ratios’ 95% confidence intervals for two-sided *χ*^2^ tests and fixed-effect meta-analysis. Individual studies (black) and meta-analyses (red). * Indicates new replication data sets. Numbers of cases/controls in parenthesis

Figure 2 shows the confidence intervals for the ORs of both the individual samples and the three different meta-analyses. The protective effect of the variant is evident with the upper bound of all confidence intervals at or below 1, with the exception of the smaller Danish BD sample.

### BrainSpan developmental transcriptome

**Fig. 3:**
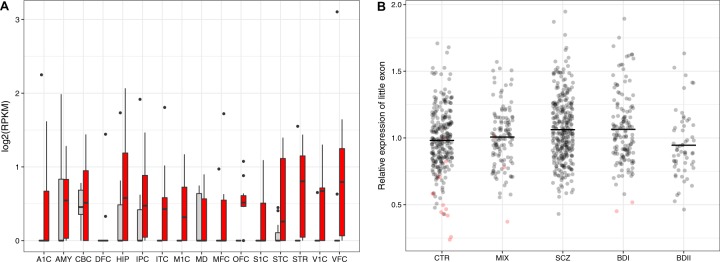
Alt-2 splice pattern expression at different development stages and by diagnosis. **a** Expression of the “little” exon in 16 different brain regions for individuals aged 0–10 years old (gray) and individuals aged 11 and older (red) from the BrainSpan database. The median is indicated by a horizontal black bar, but is not always visible as the median is zero in several brain regions. Brain region abbreviations and formal statistical tests of differential expression between age groups in table S4. **b** Alt-2 expression in blood measured by qPCR in the Norwegian sample. Carriers of the minor allele at rs41283526 in red, non-carriers in black. Mean for each diagnostic group (black horizontal bar). See Table 2 for statistical tests

Most exons of *ANK3* are expressed at all development stages and in all brain regions whereas the “medium” exon ramps in expression through childhood and the “little” exon is switched on in early adolescence^18^. A Wilcoxon test of higher expression of the “little” exon in those over 10 years old across all brain regions was highly significant (*p*-value = 1.386e−08). In order to determine whether this temporal pattern is consistent between brain regions, we plot the expression level of the little exon in infants and children vs. adolescents and adults for each brain region (Fig. 3a). This shows that in 10 of 16 regions the expression in those under 10 years old has a median of 0 while it is greater than zero for those older than 10 years old. Formal testing for differential expression (Table S4) shows that for six of these regions, the higher expression in adolescents and adults is nominally significant at 5% (three remain significant after correction for multiple testing). This confirms the unique developmental expression pattern of the “little” exon.

### Differential expression in blood

Expression levels of the alt-2 splice pattern in blood cells were compared across diagnostic groups (Fig. 3b). Carriers of the rs41283526*G allele are concentrated in the control group and have an expression mostly in the lowest percentile, graphically demonstrating the protective effect of the loss-of-function allele. Mean expression is statistically significantly higher in the SCZ and BDI groups, whereas it is not for the BDII and MIX groups (Table 2). Crucially, these tests remained significant when we excluded carriers of rs41283526*G, thus indicating that the differential expression of the alt-2 splice pattern is not solely driven by rs41283526*G.

Figure 3b shows that the SCZ and BDI groups have an excess of very high expressors relative to the control group, and we therefore performed a Fisher association test between high expression (defined as above 1.5) and diagnostic group. We find that the BDI group has the highest odds ratio at 4.9 (*p*-value = 2.0E–03) (Table S11).

### *ANK3* isoforms in human brain

**Fig. 4:**
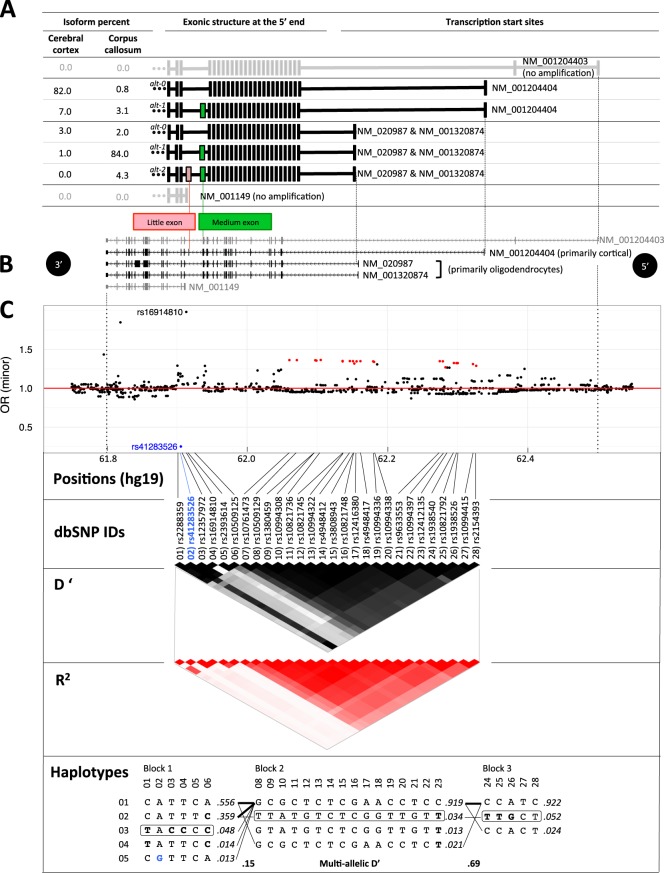
Expression of the *ANK3* isoforms and its relationship to the genetic risk for bipolar disorder. **a** Summary statistics of isoform expression levels in two different brain regions by Pacific Biosciences single-molecule real-time sequencing. Percentages do not sum to 100% due to the high number of very low frequency isoforms not represented in the figure. **b** *ANK3* transcript models as listed by the RefSeq database. Note that our long read data summarized in **a** show the RefSeq models to be incomplete and possibly incorrect. **c** Summary statistics from the PGC Bipolar Disorder GWAS (2011) in the *ANK3* region. Odds ratios for the minor allele with genome-wide significantly associated SNPs in red (*p*-value < = 5.0E–08). Rs41283526 odds ratio from Norwegian sample added to plot (in blue) for consistency with lower panels. *r*^2^ and *D*’ were computed from the Norwegian sample including all samples irrespective of diagnostic group. Haploblocks were computed in Haploview: significant SNPs from Table 3 highlighted in bold and minor haplotypes with majority of significant SNPs in black rectangles. Haplotype frequencies are highlighted in italics. Haplotypes are connected by a thin line if >1% and by a thick line if greater than 10%

We established that the alt-2 splice pattern is most strongly expressed in the corpus callosum by PCR amplification of cDNA generated from total RNA of seven different regions of human brain (Table S7). The RefSeq models show the alt-2 splice pattern being expressed from the TSS of NM_001204404 (Fig. 4b). However, because several kilobases of constitutive exons separate the genetic ROI from the TSS, we considered it necessary to use long read sequencing to clearly establish from which TSS each of the alternative splice patterns were expressed.

We determined that only the TSSes of NM_001204404 and NM_020987 are active in the seven brain regions that we tested (Table S8). Our Pacific Biosciences sequencing data from human brain identified five isoforms transcribed from these TSSes (Fig. 4a), one of which incorporates the alt-2 splice pattern and is transcribed from the TSS of NM_020987 (at 62.2 Mbp). The TSS of NM_020987 is dominant in the corpus callosum. The TSS of NM_001204404, on the other hand, is clearly dominant in the cerebral cortex. In terms of the genetic ROI, alt-0 is clearly dominant in cerebral cortex (82%), whereas alt-1 is the dominant isoform in corpus callosum (84%). As to alt-2, it is only detected in corpus callosum and transcribed from the NM_020987 TSS.

### Differential expression and bipolar disorder associated SNPs

We have demonstrated that (1) rs41283526*G has protective and statistically significant effect in multiple independent samples; (2) BD and SCZ patients have elevated expression of the alt-2 splice pattern in blood (even when excluding the effect of rs41283526*G); and (3) the alt-2 splice pattern is transcribed from the TSS of NM_020987 which is the dominant TSS in corpus callosum. We then sought to establish how these results relate to the existing GWAS results (Fig. 4c) and in particular to the PGC BD GWAS^7^. There are 22 genome-wide significant SNPs in the PGC BD phenotype association study located in *ANK3*. They are all in strong linkage disequilibrium, with an OR of ~1.3 and located within ~100 kbp of the TSS of NM_020987. There are no significant SNPs upstream of the TSS of NM_001204404, which suggests that the significant SNPs may control the regulation of an *ANK3* isoform transcribed from the TSS of NM_020987. For each of 28 SNPs (22 from PGC plus the SNP with the highest OR, rs16914810, and 4 SNPs in strong LD with and proximity to rs41283526), we tested for differential expression (DE) of alt-2 in carriers vs. non-carriers of the minor allele (higher expression in carriers for all sites except rs41283526*G where we test for lower expression). As expected, the protective allele rs41283526*G stands out with a highly significant lowered expression (Table 3). The minor alleles of the other 27 sites, which confer risk of BD, are associated with higher expression of alt-2 (8 of these at 5% significance or lower). This is in complete concordance with the protective effect of rs41283526*G, which lowers alt-2 expression (Table 3). This result remains significant when we eliminate any possible effect of rs41283526*G on the DE test results by excluding carriers of rs41283526*G (Table 3). These 28 tests are not independent as the minor alleles are present on only two different haplotypes (Fig. 4), we therefore do not correct for multiple testing.

Four of the significant SNPs are located close to the “little” exon whereas four are upstream of the TSS. We therefore investigate the LD structure of the region to establish its influence on these results (Fig. 4c). This shows that there are two independent signals (one from the genetic ROI region and one from the TSS) and these are independent of rs41283526. The PGC GWAS risk alleles, that are also significantly associated with an increased expression of alt-2, are located on the minor haplotypes in different blocks: haplotype 03 in block 1 (ROI) and haplotype 02 in block 3 (TSS). Interestingly, these two minor haplotypes do not form part of the same haplotype across blocks as can be seen from the haplotypes in Fig. 4c and the very low *r*^2^ and *D*’ between the tag SNPs 03 (rs12357972) and 26 (rs1938526). Moreover, these risk haplotypes are independent of rs41283526*G which is located on its own haplotype 05 in block 1 which extends into the major haplotype 01 in block 02 and 03.

We did also perform a classical conditional regression analysis for rs41283526 for the BD, SCZ, and BD & SCZ diagnostic groups in the Norwegian sample (unpublished) as a second approach to studying the relationship between the BD GWAS SNPs and rs41283526. However, due to the modest sample size, none of the genome-wide associated hits from PGC BD GWAS showed significant association in the logistic regression (*p*-values > 0.05 in all three diagnostic groups), so the logistic regression with conditioning on rs41283526 was not informative. This lead us to focus on the differential expression results presented above, where informative results were obtained.

## Discussion

BD is a polygenic, and most probably also a heterogeneous disease, making it difficult to isolate and characterize the complex underlying molecular mechanisms^5^. Here, we present multiple statistically significant findings of the involvement of *ANK3* at the DNA and RNA levels, which are consistent with each other and with existing GWAS data, and inform on the underlying molecular genetic mechanisms of BD.

### The protective effect of rs41283526*G

We report replication of our previous findings, confirming the protective effect of rs41283526*G against BD and SCZ despite the fact that it is difficult to achieve statistical significance for low frequency SNPs^27^. In particular, six of seven individual cohort one-tailed tests are significant at 5% significance (Table 1A). In addition, the meta-analyses (weighted *Z*-score) for BD (*p*-value: 6.8E–05) and SCZ (*p*-value: 8.2E–04) are both significant.

The effect sizes reported, although modest in the context of statistical testing, are strong in the context of the BD GWAS literature. Even the weakest effect size observed in our data (Swedish OR = 0.72) rivals that of the strongest risk effects previously reported^6,7^, while the Norwegian effect (OR = 0.25) is several times stronger. However, the range of ORs is wide for BD [0.25,0.72] and for SCZ [0.15,0.81], thus making it difficult to estimate the true strength of the protective effect. There are two factors that are most likely to be driving the variance in ORs: the low frequency of rs41283526*G (Table 1A) and study heterogeneity, which tends to affect the analysis of complex phenotypes such as mental-health-related diseases^28^. However, this variance does not detract from the strong and statistically significant protective effect

The protective effect of a loss-of-function variant may be somewhat counter-intuitive, because one typically thinks of loss-of-function as being deleterious. This is correct when considering a variant in isolation; however, if one considers the wider genetic context in which other variation may lead to pathologically high expression levels, it then becomes clear how a loss-of-function allele can restore balance. The high-effect size may also be surprising, but this is explained by the total loss-of-function of the splice site for the affected allele^18^. Finally, it may also seem illogical that dysregulation of a minor isoform of *ANK3* could be the underlying cause of the gene’s association with the disorder. However, the 50% change in expression observed in heterozygote carriers^18^ is as likely to have physiologic effects, as similarly sized changes to the other more abundant isoforms, because it is the relative dosage of proteins that is the key.

### Expression of the alt-2 splice pattern

Ideally we would have obtained RNA samples from the brains of the cases and controls that we had genotyped. For obvious reasons, this was not possible, so we had to rely on blood samples. We know that the absolute levels of *ANK3* expression are much lower in blood than in brain. However, as long as rank order of expression is somewhat maintained (i.e., that a high expressor in brain does not become a low expressor in blood), then we expect to be able to detect significant differences between groups, if our main hypothesis is correct.

The mean expression of alt-2 is significantly elevated in SCZ and BDI relative to controls (Fig. 3b) and, crucially, this is also true when carriers of rs41283526*G are excluded (Table 2). This indicates that the observed elevated expression of alt-2 is not solely driven by the rare rs41283526*G allele and that elevated expression is a risk factor in the general patient population. The concordance between SCZ and BDI is perhaps not surprising given that they are the two psychiatric diseases with the highest genetic correlation^29,30^ and are both characterized by psychotic symptoms.

The test of differential expression of alt-2 between those over and under 10 years old (Brainspan data set) shows that expression of the isoform begins in adolescence (Fig. 3b; Table S4). This provides further corroborative evidence of this isoform’s central role in the etiology, because the onset of the first hypomanic episode of BD typically happens in adolescence^1^ and no other exon of *ANK3* shows such a change in expression during post-natal development according to the BrainSpan database.

The function of the evolutionarily conserved peptide sequence encoded by the little exon is unknown. However, available bioinformatic structural data suggests that the peptide sequence is unstructured linker possibly containing a linear motif. The most significant hit in the Eukaryotic Linear Motif database^31^ for the little exon peptide sequence (GNRCTWYKIPKVQEFTVK) is for an N-myristoylation site which mediates myristate attachment to an N-terminal glycine residue. In *ANK3*, this glycine residue is not N-terminal; however, post-translational myristoylation is known to typically occur following a caspase cleavage event resulting in the exposure of the internal glycine residue^32^. The C-terminal amino acids of the “medium” exon followed by this glycine residue is a possible match for a caspase cleavage site^33^. Further, the extended model of the myristoylation motif is 17 amino acids long^34^, which is a close match to the 18 amino acids of the “little” exon. Interestingly, myristate attachment to an N-terminal glycine residue acts as a membrane anchor for some soluble cytoplasmic proteins^32^.

### Indications of *ANK3* isoforms cell-type specificity

Our long read sequencing of *ANK3* transcripts shows that the RefSeq list is not complete, since it contains only one of the five isoforms that we detect, namely the most abundant isoform in the corpus callosum (alt-1 isoform with the NM_020987 TSS). Further, RefSeq suggests that the NM_001204404 isoform incorporates the alt-2 pattern, but our data show only the alt-0 and alt-1 splice patterns being expressed from this TSS.

The alt-2 splice pattern is incorporated into the isoform transcribed from the TSS of NM_020987 and is expressed in the corpus callosum, but not in the cerebral cortex (Fig. 4a). The TSS of NM_020987 is dominant in the corpus callosum which consists primarily of myelinated axons but where the cell soma are almost exclusively oligodendrocytic^35^. Thus, our data are consistent with the existing data obtained by conditional knock-out of *ANK3* in mouse oligodendrocytes^14^, as they also show that the NM_020987 TSS is dominant in oligodendrocytes. The alt-2 splice pattern is not incorporated into the isoforms transcribed from the TSS of NM_001204404, which is dominant in the cerebral cortex. Axons in the cerebral cortex, and particularly the sections in layers I–III have been shown to not be myelinated^36^, thus sequence from this brain region provides a better indication of the RNA content of neurons. In summary, our results suggest that alt-2 is expressed in oligodendrocytes, but probably not in neurons.

It has previously been hypothesized that BD and SCZ may be caused by disruption of the myelination process^37,38^. There has been some suggestive evidence of molecular abnormalities at the nodes of Ranvier^39^, but perhaps the most compelling evidence of this comes from brain imaging data which shows the smaller size of the corpus callosum in both BD patients and their first degree relatives^40,41^, and reduced levels of myelin^42–44^. Our results indicate that, at least with regards to *ANK3*’s role in the etiology of BD, the elevated expression of the alt-2 splice form is most likely occurring in oligodendrocytes and probably not in neurons, and its protein product may well be located at the nodes of Ranvier since an *ANK3* isoform has been detected on the glial side of the paranodes (Fig. 1c)^14^.

It is important to note that we are not suggesting that the corpus callosum is necessarily the brain structure where the pathology originates. Rather it is here that both the molecular and imaging phenotypes are clearly detectable due to the high levels of myelinating oligodendrocytes. Myelinated axons are found throughout the brain and the “little” exon is also detected in most brain structures after 10 years of age (Fig. 3a), thus our data provide no strict indication of the brain region in which the imbalance may be causing the BD behavioral phenotype.

### Relationship with GWAS results

Our analysis of the differential expression of alt-2 between carriers and non-carriers of the PGC GWAS minor alleles demonstrates complete consistency between the genotype/phenotype association of the PGC and the genotype/isoform expression association we present in Table 3 and Fig. 4b: risk minor alleles from the PGC study are associated with an elevated expression of alt-2, while the protective minor allele of rs41283526 is associated with a halving of alt-2 expression. Further, the haplotype structure reveals that there are two independent minor haplotypes raising the expression of alt-2 that are independent of rs41283526*G: the minor haplotype 03 in block 1 (ROI) and the minor haplotype 02 in blocks 2 and 3 (TSS). Interestingly, with the benefit of hindsight, it is possible to see that the PGC OR plot contains tell-tale signs of our findings (Fig. 4c): (1) the PGC significant SNPs frame the TSS of NM_020987 and, (2) the genetic ROI is almost devoid of SNPs with a neutral OR, while the rest of the *ANK3* gene is dominated by SNPs with ORs of 1.

## Conclusion

The loss-of-function allele rs41283526*G has a strong protective effect against both BD and SCZ that replicates in six independent samples. It is not itself a major contributing factor to the association of *ANK3* with BD (due to its low frequency), but it strongly suggests that elevated expression of the *ANK3* isoform incorporating exon ENS00001786716 may be a factor in the pathobiology of the disorder. Our analysis of human RNA data sets shows that the available data strongly support this hypothesis and previous GWAS findings are also consistent with it. Further, we provide evidence that the specific isoform is most strongly expressed in corpus callosum, which suggests that it may be in oligodendrocytes that the imbalance occurs. We look forward to future studies of the role of this specific isoform in myelination and, particularly, in the formation and maintenance of nodes of Ranvier.

## Electronic supplementary material

Supplementary material
