# Supplementary material for: Elevated expression of a minor isoform of ANK3 is a risk factor for bipolar disorder
Source: Transl Psychiatry. 2018 Oct 8;8:210. doi: 10.1038/s41398-018-0175-x (PMC6175894; doi:10.1038/s41398-018-0175-x)
Supplement: Supplementary file 1 — Supplementary material [file 41398_2018_175_MOESM1_ESM.docx]

**Supplementary materials**

**Association replication and meta-analysis**

**Table S1: Hardy-Weinberg equilibrium test statistics for all samples performed in PLINK**

**Table S2: 1000 genomes (phase 3) allele frequencies with sub-population detail and second independent Norwegian control population.**

**Table S3: Recruitment, genotyping and phenotyping details for all samples and relevant references**

**Brainspan developmental transcriptome**

**Table S4: Statistical test of the increase in the expression of the little exon after age 10 across brain regions**

Wilcox one-sided test of greater expression of the “little” exon in individuals older than 11 years old (vs. those under 10 years old), in 16 different brain regions (Brainspan data). Some developmental stages and brain structures have small sample sizes, so we excluded any brain structure for which fewer than five individuals had been sampled and we excluded any developmental stage with fewer than three brain structures in the data set.

**Differential expression in blood**

RNA Extraction and cDNA Generation

Blood samples for gene expression analysis were collected using Tempus Blood RNA Tubes (Life Technologies Corporation, Carlsbad, CA, USA), and stored at -80°C until analysis.

Total RNA was extracted either automated with the ABI PRISM 6100 Nucleic Acid PrepStation (Life Technologies Corporation, Carlsbad, CA, USA) and the Tempus 12-port RNA Isolation Kit or manually with the Tempus Spin RNA Isolation Kit (Life Technologies Corporation, Carlsbad, CA, USA) according to the manufacturer’s protocol. RNA quantity was determined using a NanoDrop 8000 Spectrophotometer (NanoDrop Technologies, Wilmington, DE, USA).

cDNA was generated from 1 μg of total RNA, using the High-Capacity cDNA Reverse Transcription Kit (Life Technologies Corporation, Carlsbad, CA, USA) according to the manufacturer’s protocol.

Quantitative RT-PCR

RT-qPCR was performed on the ABI PRISM 7900HT Real-Time PCR System (Life Technologies Corporation, Carlsbad, CA, USA), using a SYBR green assay. Each sample was assayed in triplicate with one ANK3 “little” exon specific primer set (5’-CCAATGCCCCTGAAATGCTC-3’ and 5’-CCGTAAACTCTTGTACCTTGGG-3’), and normalized to the gene expression of a primer pair upstream in the ANK3 transcripts (5’-GCTTGATCGAGCTGCCCCCA-3’ and 5’-GCTGGAGGAGAAGCTGGACGC-3’). Briefly, 10 ng of cDNA was used as input in each PCR reaction, which consisted of 1X Power SYBR Green Master Mix (Life Technologies Corporation, Carlsbad, CA, USA), 1 µM of the ANK3 “little” exon specific primers, or 0.3 µM of the upstream primers. After an initial heating step at 95°C for 10 min to activate the polymerase, 40 PCR cycles were performed. Each cycle consisted of a denaturation step at 95°C for 15 s, followed by a combined annealing and extension step at 60°C for 1 min. A melt curve analysis was performed after each PCR, to assess the specificity of the assay. Using qBasePLUS software (v2.6), the delta-delta-Cq model was used to determine relative target gene expression.

Sample demographics

**Table S5: Demographic information for the RNA samples**

**ANK3 isoforms in human brain**

**Brain regions with “little” exon expression**

The seven pools from different brain regions are summarized in the following table.

**Table S6: Normal human brain total RNA samples from Clontech (Mountain View, CA, USA).**

We designed primers in the “5’ constitutional” exon (5’-FAM-ACTGTCACAGAGAAGCACAA- 3’) and in the “3’ constitutional” exon (5’-TGCAGGCAGGGAATCATCAC-3’). PCR amplification was performed in 10 μl volume, using 10 ng of cDNA as input. Each PCR reaction consisted of .25 U of AmpliTaq Gold 360 (Life Technologies Corporation, Carlsbad, CA, USA), 1.5 mM MgCl2, .2 mM each dNTP, and 1 μM of each primer. After an initial heating step at 95°C for 10 min to activate the polymerase, 25 PCR cycles were performed. Each cycle consisted of a denaturation step at 95°C for 30 s, an annealing step at 60°C for 30 s, followed by an extension step at 72°C for 30 s. After cycling, there was a final extension step at 72°C for 7 min. PCR amplifications were performed on all seven brain samples described above.

Following amplification, one microliter of the amplified products were mixed with 12 μl Hi-Di Formamide (Applied Biosystems) and 2 μl Genescan 500 LIZ dye Size Standard (Applied Biosystems), and subjected to capillary electrophoresis on the Applied Biosystems 3730xl DNA Analyzer (settings: 10 ng cDNA input, 25 cycles, 10 seconds injection). The result files were analyzed in the GeneMapper 5 software (Applied Biosystems), see Table S6.

**Table S7: Peak height in units of fluorescence from fragment analysis of labeled PCR product spanning “little” and “medium” exons (Only reporting peaks above background level).**

**Active transcription start sites**

Full-length cDNA was generated from 1 µg of total RNA, using the SMARTer® PCR cDNA Synthesis Kit (Takara Bio USA, Inc., Mountain View, CA, USA). Each reaction was setup on ice. For each sample, 1 µl total RNA, 1 µl 3’ SMART CDS Primer II A (12 µM), and deionized water to a final volume of 4.5 µl was combined in separate 0.2 ml reaction tubes. The tubes were incubated at 72°C in a hot-lid thermal cycler for 3 min, followed by 42°C for 2 min. Keeping the samples in the thermal cycler, 5.5 µl first-strand master mix was added. This consisted of 2 µl First-Strand Buffer, 0,25 µl DTT (100 mM), 1 µl dNTP Mix (10 mM), 1 µl SMARTer II A Oligonucleotide (12 μM), 0.25 µl RNase inhibitor, and 1 µl SMARTScribe Reverse Transcriptase (100 U). The samples were mixed by pipetting, spun down, and incubated at 42°C for 90 min, followed by reaction termination at 70°C for 10 min, and allowed to cool to 4°C in the thermal cycler. Finally, 40 µl TE buffer (10 mM Tris [pH 8.0], 0.1 mM EDTA) was added, for a total of 50 µl first-strand products.

Target-specific products were generated directly from first-strand cDNA products, using the QIAGEN LongRange PCR Kit (QIAGEN, Hilden, Germany). Each amplification was setup on ice, and carried out in separate wells in a 96-well plate. For each sample, 5 µl first-strand products was combined with a master mix consisting of 5 µl LongRange PCR Buffer, 10X, 2.5 µl dNTP mix (10 mM each), 0,4 µM of each primer (see table?), 0,4 µl LongRange PCR Enzyme Mix, and RNase-free water to a total volume of 50 µl. A hot-lid thermal cycler was pre-heated to 93°C, the plate loaded and then subjected to an initial denaturation and activation hold at 93°C for 3 min. Then, 10 cycles of denaturation at 93°C for 15 s, annealing at 60°C for 30 s, and elongation at 68°C for 13 min was followed by a further 28 cycles of denaturation at 93°C for 15 s, annealing at 60°C for 30 s, and elongation at 68°C for 13 min with a 20 s time increment per cycle. Total cycling time was about 9 hours. Products were analyzed with the Agilent DNA 12000 Kit on the 2100 Bioanalyzer system (Agilent Technologies, Santa Clara, CA, USA) and detected peaks are reported in the table below.

**Table S8: Product sizes for 4 different full-length amplifications of ANK3.**

Forward primers are placed in the first exon of each of the known ANK3 transcripts (as defined in RefSeq).

**Pacific Biosciences sequencing**

Full-length cDNA generation was performed on corpus callosum and cerebral cortex samples as described in the previous section. Target-specific PCR was performed using the forward primers in the first exon of NM_001204404 (5’-GCACCCGTAAATGGACTTTGG-3’) and of NM_020987 (5’-GCCTGATTAGAGGTGCCTGA-3’), and a reverse primer three exons downstream of the region of interest (5’-CTAAGGTGTCTGCAGCCCAG-3’).

Target-specific products were generated directly from first-strand cDNA products, using the QIAGEN LongRange PCR Kit (QIAGEN, Hilden, Germany). Each amplification was setup on ice, and carried out in separate wells in a 96-well plate. For each sample, 5 µl first-strand products was combined with a master mix consisting of 5 µl LongRange PCR Buffer, 10X, 2.5 µl dNTP mix (10 mM each), 0,4 µM of each primer, 0,4 µl LongRange PCR Enzyme Mix, and RNase-free water to a total volume of 50 µl. A hot-lid thermal cycler was pre-heated to 93°C, the plate loaded and then subjected to an initial denaturation and activation hold at 93°C for 3 min. Then, 35 cycles of denaturation at 93°C for 15 s, annealing at 60°C for 30 s, and elongation at 68°C was performed.

Sample preparation for PacBio sequencing was performed with the SMRTbell Template Prep Kit 1.0, following recommendations in the 2 kb Template Preparation and Sequencing protocol. For each brain region, 1500 ng of PCR products were pooled on equal volumes, then purified and concentrated with AMPure ® PB magnetic beads. Following end-repair on 750 ng purified pools, blunt adapter was ligated with 15 minutes incubation. Final libraries of 120-150 ng were recovered after the final two 0.6x AMPure ® PB magnetic bead purifications. Size distribution of the libraries with main peaks 3.7-4.1 kb were confirmed with the Agilent 2100 Bioanalyzer System using the DNA High Sensitivity kit (these peaks appear larger than they really are due to the ligated non-double stranded SMRTbells). Finally, each library was sequenced on one flow cell of the Pacific BioSciences RS II (Menlo Park, CA, USA) yielding approximately 80k productive wells for each brain region (table S9).

Reads satisfying length, quality, and exonic structure filters (as described in the materials and methods) were mapped to the reference human genome (hg19) with the bbmap software (part of the BBTools software suite: http://jgi.doe.gov/data-and-tools/bbtools/) .

**Table S9: Summary of two Pacific Biosciences flow cell output after filtering out poor quality polymerase reads**

Subsets of the PacBio datasets can be browsed in Integrative Genomics Viewer (IGV):

1. Download and unzip the datasets: <http://folk.uio.no/timothyh/ank3Expression_supmat/IGVsessionPackage.zip>
2. Open IGV: <http://software.broadinstitute.org/software/igv/download> (follow the instructions for your operating system)
3. In the IGV menu: File > Open Session > select the igv_session.xml file located in the unzipped IGVsessionPackage.zip
4. This should load the data and you should see a display similar to Figure S9

Note that each dataset is down-sampled to approximately 2000 reads. As a result, these downloadable subsets no longer retain information on the relative abundances of different isoforms across brain regions or TSSs.


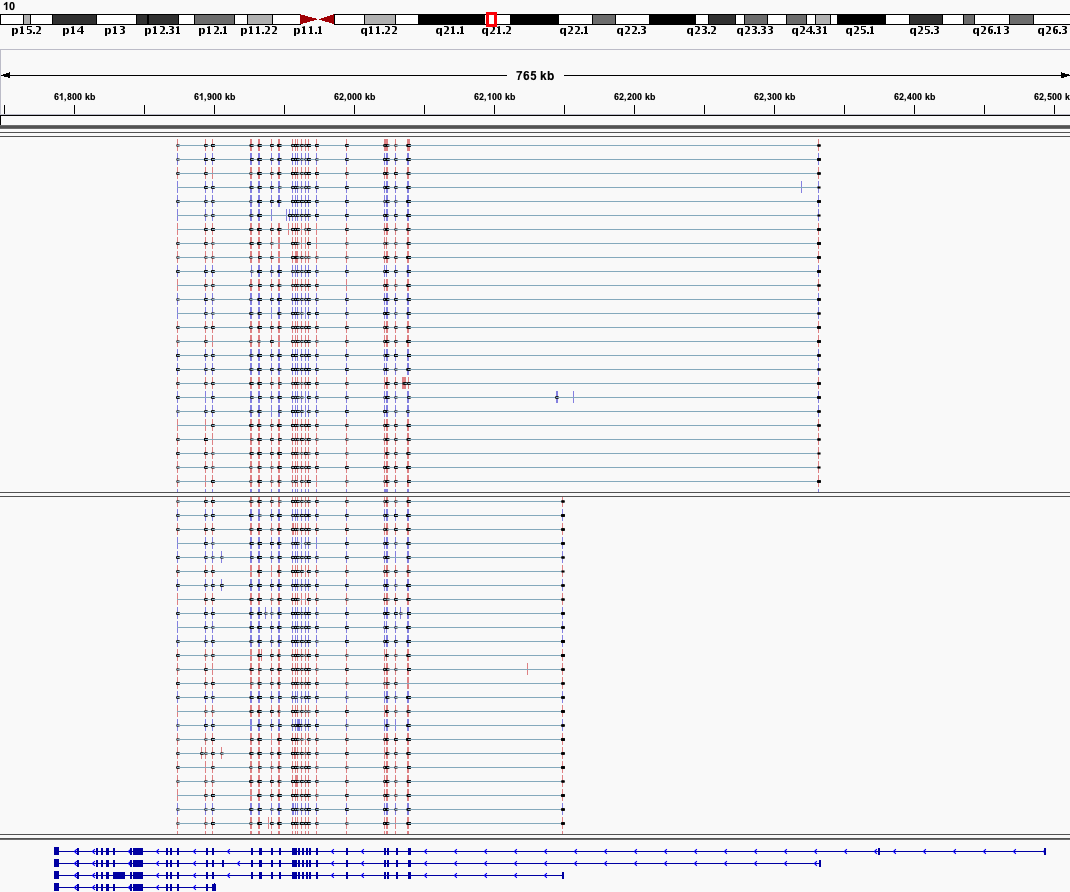


**Figure S10: Pacific Biosciences reads of corpus callosum cDNA from ANK3 TSS to ROI displayed in the Integrative Genome Viewer.**

Upper panel: reads from the NM_001204404 TSS.

Lower panel: reads from the NM_020987 TSS.

Red / blue color: reads mapping to forward / reverse strand, respectively.

**Table S11: Elevated expression of ANK3 "little" exon as a risk factor for BD and SCZ.**

One-sided Fisher test of elevated expression in cases vs. controls (elevated expression defined as relative expression greater than 1.5)
